# On Arterial Transfusion of Blood

**Published:** 1872-01

**Authors:** 


### Miscellaneous.

### On Arterial Transfusion of Blood.

Professor Hueter recorded some time ago a case of poisoning with carbonic oxide, in which he preserved the life of the patient by transfusion. More recently, in the *Centralblatt,* he has recommended the same, on the ground of the successful issue of three cases where healthy blood was injected to remove the symptoms accompanying intense septicæmia. Hueter pursued in these cases not the usual plan of injecting the venous blood of a healthy man into the *artery* of the invalid. He performed the operation in the following way: During the defibrination of the blood by beating and filtration through a fine piece of muslin by assistants, he exposes the radial artery or the tibialis posticus, above the malleolus internus, the latter being just as easy to find as the former. Any slight hemorrhage is carefully arrested, and a very small opening is made in the sheath of the artery, which is separated from the adventitia; a sound is then pushed under the artery, and moved hither and thither, till about two and a half centimetres of the artery is isolated. Four pieces of strong silk are now passed beneath the vessel, of which one forms a reserve ligature. The silk nearest to the heart is now tied tightly, so as to prevent all entrance into the vessel of blood coming from the heart. The injection syringe is now filled, and the lowermost silk slightly pulled, so as to stretch the vessel. An opening is now made in its upper part, by cutting it about half way transversely with scissors. The canula is introduced and secured by the third thread. The tension hitherto kept on the lowermost silk is now relaxed and the injection begun to be forced in. When the injection is completed, the lowermost thread is tightened, and the piece of artery between the two ligatures excised. The wound is simply dressed. The principal difficulties of the operation are its complexity, and the necessity that exists of maintaining a considerable pressure on the piston to overcome the cardiac pressure. On the latter ground Hueter recommended the employment of Mosler's injection syringe, in which the piston works with a screw. In Hueter's opinion, the objections are far outweighed by the advantages which arterial transfusion affords. One of these advantages is, that the blood reaches the heart more slowly, and with greater steadiness and regularity, than by venous transfusion. He regards the injection of small quantities (two, three or four ounces) as useless, in most instances from eight ounces to one pound being requisite. But if so large a quantity be suddenly thrown upon the heart as occurs in injection by the veins, a fatal arrest of its activity may occur. It must be remembered, also, that in consequence of the bleeding prior to the injection, as much unhealthy blood is removed as good is introduced from the system. Another advantage attendant on the arterial injection is security against the introduction of air, any small quantities that may be introduced being rapidly absorbed during the passage of the blood through the capillaries. By this method, also, all danger of phthisis is avoided, which in many instances, when transfusion of the veins would otherwise have proved successful, had led to the death of the patient. It has not yet been ascertained whether the contact of a large quantity of blood, rendered arterial by whipping with the waste of the right heart, is of any real advantage. In the mode of transfusion by the arteries, the blood necessarily becomes venous during its passage through the capillaries. In conclusion, Mr. Hueter observes that transfusion, whether through the veins or arteries, constitutes a weapon against diseases which can in no other way be contested, and points out the excellent results we may anticipate from its employment.—*Aerztliches Literaturblatt, No.* 6, 1871, and *Langenbec's Archiv*, 1870.—*Cincinnati Lancet and Observer.*

